# Prenatal Phthalate Exposure and Childhood Growth and Blood Pressure: Evidence from the Spanish INMA-Sabadell Birth Cohort Study

**DOI:** 10.1289/ehp.1408887

**Published:** 2015-04-07

**Authors:** Damaskini Valvi, Maribel Casas, Dora Romaguera, Nuria Monfort, Rosa Ventura, David Martinez, Jordi Sunyer, Martine Vrijheid

**Affiliations:** 1 Centre for Research in Environmental Epidemiology (CREAL), Barcelona, Spain; 2 Pompeu Fabra University, Barcelona, Spain; 3 CIBER de Epidemiología y Salud Pública (CIBERESP), Spain; 4 Department of Environmental Health, Harvard T.H. Chan School of Public Health, Boston, Massachusetts, USA; 5 School of Public Health, Imperial College London, London, United Kingdom; 6 Instituto de Investigación Sanitaria de Palma (IdISPa), Palma de Mallorca, Spain; 7 CIBER Fisiopatología de la Obesidad y Nutrición (CIBEROBN), Spain; 8 Hospital del Mar Medical Research Institute (IMIM), Barcelona, Spain

## Abstract

**Background:**

Human evidence on the effects of early life phthalate exposure on obesity and cardiovascular disease risks, reported by experimental studies, is limited to a few cross-sectional studies.

**Objectives:**

We evaluated the associations between prenatal phthalate exposure and childhood growth and blood pressure in a Spanish birth cohort study.

**Methods:**

We assessed exposure using the average of two phthalate metabolite spot-urine concentrations collected from the mothers in the first and third pregnancy trimesters (creatinine-adjusted, *n* = 391). Study outcomes were the difference in age- and sex-specific *z*-scores for weight between birth and 6 months of age; and repeated age- and sex-specific *z*-scores for body mass index (BMI) at 1, 4, and 7 years; waist-to-height ratio at 4 and 7 years; and age- and height-specific *z*-scores for systolic and diastolic blood pressure at 4 and 7 years.

**Results:**

The sum of five high-molecular-weight phthalate metabolites (ΣHMWPm) was associated with lower weight *z*-score difference between birth and 6 months (β per doubling of exposure = –0.41; 95% CI: –0.75, –0.06) and BMI *z*-scores at later ages in boys (β = –0.28; 95% CI: –0.60, 0.03) and with higher weight *z*-score difference (β = 0.24; 95% CI: –0.16, 0.65) and BMI *z*-scores in girls (β = 0.30; 95% CI: –0.04, 0.64) (*p* for sex interaction = 0.01 and 0.05, respectively). The sum of three low-molecular-weight phthalates (ΣLMWPm) was not significantly associated with any of the growth outcomes. ΣHMWPm and ΣLMWPm were associated with lower systolic blood pressure *z*-scores in girls but not in boys.

**Conclusions:**

This study suggests that prenatal phthalate exposure may be associated with postnatal growth and blood pressure in a sex-specific manner. Inconsistencies with previous cross-sectional findings highlight the necessity for evaluating phthalate health effects in prospective studies.

**Citation:**

Valvi D, Casas M, Romaguera D, Monfort N, Ventura R, Martinez D, Sunyer J, Vrijheid M. 2015. Prenatal phthalate exposure and childhood growth and blood pressure: evidence from the Spanish INMA-Sabadell birth cohort study. Environ Health Perspect 123:1022–1029; http://dx.doi.org/10.1289/ehp.1408887

## Introduction

Phthalates are a class of synthetic compounds widely used in the manufacture of many industrial and consumer products, such as polyvinyl chloride (PVC) products including building materials, cables and wires, clothing, and food and beverage containers and non-PVC products including adhesives, enteric-coated capsules, and personal-care articles (Wittassek et al. 2011). High-molecular-weight phthalates (HMWP), such as di(2-ethylhexyl) phthalate (DEHP), have been commonly used in the manufacture of PVC applications, and low-molecular-weight phthalates (LMWP), such as diethyl phthalate (DEP), are commonly used in non-PVC personal care products. Food consumption—especially of packaged food and beverages—is considered to be the main route of human exposure to HMWP, and the use of personal-care products and indoor air may contribute importantly to LMWP exposure (Adibi et al. 2008; Koch et al. 2013; Wittassek et al. 2011). Phthalates are nonpersistent compounds that are quickly metabolized in the human body and excreted in urine (within few hours or days after exposure) (Wittassek et al. 2011). Studies in many countries have reported detectable levels of phthalate metabolites in urine of almost all individuals tested (> 90%), including pregnant women (Adibi et al. 2008; Braun et al. 2012; Cantonwine et al. 2014; Casas et al. 2011). Detectable levels of phthalate metabolites have been also found in amniotic fluids (Jensen et al. 2012) and cord blood (Yan et al. 2009), indicating fetal exposure.

Evidence from experimental studies suggests that phthalate exposure in early life may disrupt developmental processes and, potentially through permanent epigenetic changes, may lead to increased risk of cardiometabolic diseases later in life (Barouki et al. 2012). *In vitro* and *in vivo* studies have shown that phthalates and phthalate metabolites are thyroid hormone and androgen antagonists and may activate the peroxisome proliferator–activated receptors (PPARs), a nuclear receptor superfamily with a key role in adipogenesis, lipid accumulation, and insulin resistance (Boberg et al. 2008; Hao et al. 2012; Taxvig et al. 2012). These mechanisms suggest a potential link to the development of obesity and diabetes. More recently a potential link between phthalate exposure and cardiovascular disease (CVD) risk has emerged, almost exclusively based on cross-sectional data (Trasande et al. 2013b, 2014). Phthalate metabolites are shown to induce the release of proinflammatory cytokines *in vitro* (Jepsen et al. 2004), and phthalate metabolite concentrations were positively associated with urine markers of oxidative stress during pregnancy in a cohort of Puerto Rican women (Ferguson et al. 2014). Further, in two recent cross-sectional studies of children who participated in the National Health and Nutrition Examination Survey (NHANES), phthalate urine concentrations were associated with higher systolic blood pressure (BP) (Trasande et al. 2013b) and low-grade albuminuria, a marker of vascular dysfunction associated with chronic kidney and CVD risks (Trasande et al. 2014). Thus, we evaluated the associations between prenatal exposure to several phthalates and growth outcomes [i.e., weight gain, body mass index (BMI), waist-to-height ratio] and systolic and diastolic BP in the first 7 years of life using data from a prospective birth cohort study in Spain. Because child rapid weight gain in the first months after birth has been consistently associated with increased risk of obesity later in childhood and adulthood (Druet et al. 2012), we further examined whether phthalate exposure is associated with weight gain in the first 6 months of life.

## Methods

*Population and data collection*. The Spanish population-based birth cohort study INMA (“Infancia y Medio Ambiente”—Environment and Childhood) recruited 657 women in the first trimester of pregnancy in the two primary health care centers of Sabadell in 2004–2006 (Guxens et al. 2012). The inclusion criteria were age at least 16 years, intention to give birth in the reference hospital, no problems in communication, singleton pregnancy, and no assisted conception. The mother–child pairs were later followed in the third trimester of pregnancy, at delivery, and at child ages 6 months and 1, 4, and 7 years (participation rate in last follow-up, 76%). We analyzed 391 mother–child pairs with available phthalate and creatinine determinations measured in two spot-urine samples collected in the first (mean ± SD, 13.4 ± 1.7 gestational weeks) and third (mean ± SD, 34.1 ± 1.4 weeks) pregnancy trimesters.

Interview-based questionnaires collected information on parental sociodemographic characteristics and other maternal characteristics including medical history and lifestyle habits. In the first pregnancy trimester visit, we measured maternal height, and the mothers reported their prepregnancy weight and paternal weight and height. In the first and third pregnancy trimesters, maternal diet was assessed using a 101-item food frequency questionnaire (FFQ) validated for use in Spanish adults (Willett and Stampfer 1986) and physical activity using a previously validated short self-administered questionnaire (Norman et al. 2001). Infant feeding practices and child diet (using a 101-item FFQ) and sedentary activities were reported in postnatal questionnaires.

All mothers signed an informed consent. This study was approved by the ethics committee of the Hospital del Mar Medical Research Institute and conducted according to principles of the Helsinki Declaration (World Medical Association 2013).

*Phthalate metabolite urine concentrations*. Maternal urine samples were collected during pregnancy in 100-mL polypropylene containers and were afterwards aliquoted in 10-mL polyethylene tubes and stored at –20°C until phthalate analyses were carried out in the Bioanalysis Research Group at the Hospital del Mar Medical Research Institute (Barcelona, Spain). We measured total urine concentrations (free plus glucuronoconjugated species) in first and third pregnancy trimesters of MBzP (mono-benzyl phthalate), MEHP [mono(2-ethylhexyl) phthalate], MEHHP [mono(2-ethyl-5-hydroxyhexyl) phthalate], MEOHP [mono(2-ethyl-5-oxohexyl) phthalate], MECPP [mono(2-ethyl-5-carboxy-pentyl) phthalate], MEP (monoethyl phthalate), MiBP (monoisobutyl phthalate), and MnBP (mono-*n*-butyl phthalate). Analytical methods involved urine sample preparation with enzymatic hydrolysis with β-glucuronidase enzymes and solid-phase extraction, and analysis by ultraperformance liquid chromatography coupled to tandem mass spectrometry (Waters Corp.) and have been detailed previously (Valvi et al. 2015). The limit of detection (LOD) values were 0.5 μg/L or 1 μg/L, depending on the metabolite. All metabolites had quantified concentrations in almost all samples analyzed (values below LOD ≤ 1%). Urine creatinine concentrations were measured at the Echevarne laboratory of Barcelona (Spain) using the Jaffé method (kinetic measurement, compensated method) with Beckman Coulter^©^ reactive in AU5400 (IZASA®).

We substituted phthalate metabolite values below LOD by LOD/2. We calculated the molar sums of individual metabolites (in micromoles per liter) of DEHP because they occur from the same parent phthalate, and also of HMWP and LMWP, as they represent similar sources of exposure and they are moderately to highly correlated (ΣDEHPm, ΣHMWPm, and ΣLMWPm, hereafter) (Valvi et al. 2015). The ΣDEHPm included the simple monoester MEHP and the secondary oxidized metabolites MEHHP, MEOHP, and MECPP. The ΣHMWPm included the ΣDEHPm metabolites and MBzP. The ΣLMWPm included MEP, MiBP, and MnBP. We expressed the molar sums in nanograms per milliliter by multiplying ΣDEHPm and ΣHMWPm with the molecular weight of MEHP, and ΣLMWPm with the molecular weight of MEP, to facilitate comparisons, similar to methods followed in other studies (e.g., Wolff et al. 2010). Concentrations of individual and summed metabolites were divided by urine creatinine levels (micrograms per gram creatinine) to control for urine dilution.

All phthalate metabolite concentrations had poor reproducibility between the two pregnancy trimester [intraclass correlation coefficients < 0.25 (Valvi et al. 2015)]. Therefore, we estimated associations with the average concentration for the two trimesters, rather than single spot-urine concentrations, to better approximate average phthalate exposure during pregnancy. Phthalate metabolite concentrations were log_2_-transformed to normalize the right-skewed distributions and analyzed continuously and categorically using tertile cutoffs.

*Growth and BP outcomes*. Repeated weight measurements from birth to 6 months of age were extracted from the medical records. We used the second-order Reed sex-specific early infancy growth models to predict the weight of children without weight measurement available within ±14 days of their exact 6-month anniversary (*n* = 60), as described previously (Valvi et al. 2013). Weight gain was defined as the difference in age- and sex-specific *z*-scores for weight between 6 months and birth using the World Health Organization (WHO) referent (de Onis et al. 2009). Children with a weight *z*-score difference > 0.67 SD were classified as rapid growers (Monteiro and Victora 2005). Child weight and height were measured at 1, 4, and 7 years of age using standard protocols, without shoes and in light clothing. We calculated BMI (weight/height^2^) and used the WHO referent to estimate age- and sex-specific BMI *z*-scores (de Onis et al. 2007, 2009). Overweight was defined as a BMI *z*-score ≥ the 85th percentile. Waist circumference at 4 and 7 years of age was measured in standing position at the midpoint between the lowest rib margin and the iliac crest after a gentle expiration. We divided child waist circumference by height to calculate the waist-to-height ratio. Central obesity was defined as a waist-to-height ratio > 0.50, because this value has been reported to predict later risk for cardiometabolic syndrome in children as in adults (Browning et al. 2010; Graves et al. 2014).

Systolic and diastolic BP was measured by specially trained personnel of the research team at the child’s age 4 years at the primary health center and at 7 years at school. A digital automatic monitor (OMRON 705 CPII) and a special cuff adjusted to the upper right arm size of the children at each age were used. Measurements were taken once after at least 5 min in resting position. BP measurements may vary according to child age, height, and sex; therefore, we calculated age- and height-specific BP *z*-scores using the population mean separately in girls and boys. For this, age was grouped in 3-month intervals and height in centimeter units. High systolic and diastolic BP was then defined as a BP *z*-score ≥ the 90th percentile.

*Statistical analyses*. Generalized additive models (GAMs) assessed the linearity of the associations between the phthalate metabolite concentrations and the outcome variables. Linearity was assumed if the *p*-gain defined as the difference in normalized deviance between the GAM model and the linear model for the same exposure and outcome (Royston and Ambler 1998) was > 0.10. We used generalized estimating equations (GEEs) with an unstructured correlation matrix and a Gaussian or Poisson family specification (for continuous and dichotomous outcomes, respectively) to assess the associations between phthalate metabolite concentrations and repeated growth and BP outcome measurements. GEE models included an interaction term between the exposure variable and child age at examination. Child age at examination was included in the interaction term categorically (1, 4, and 7 years in the models for BMI; 4 and 7 years in the models for BP and waist-to-height ratio). The associations with weight gain (continuous) and rapid growth (dichotomous) in the first 6 months of life were assessed using linear regression and generalized linear models, respectively. Because phthalate effects may be sex specific (Boberg et al. 2008; Feige et al. 2010; Hao et al. 2012), we evaluated effect heterogeneity by introducing in the models interaction terms between the exposure variable and sex and by stratifying models according to sex. Statistical significance was defined by an alpha level of 0.10 for interaction terms and of 0.05 for all other effect estimates.

We selected the covariates retained in the final models using a combined approach of directed acyclic graphs (DAGs) and change-in-estimate procedures (Evans et al. 2012). The initial DAGs included maternal determinants of phthalate metabolite concentrations in this population: country of origin, education, social class [coded based on occupation using the International Standard Classification of Occupations (ISCO)–88 system], prepregnancy BMI, smoking, frequency of organic food consumption, and use of bleach during pregnancy (Valvi et al. 2015). Other covariates were included based on previous literature: maternal age at delivery, parity, gestational weight gain (Casas et al. 2013a), gestational diabetes (self-reported; yes, no), maternal physical activity (in metabolic equivalents per hour per day), alcohol consumption (yes, no), maternal urine BPA concentrations during pregnancy (Valvi et al. 2013), paternal BMI, birth weight, exclusive breastfeeding duration (Guxens et al. 2011), and child’s dietary habits [i.e., fast-food (< once/week, ≥ once/week) or sugar-sweetened beverage (< once/month, once to four times/month, > four times/month) consumptions and total caloric intake (kilocalories/day)] and time spent watching TV or playing videogames (≤ 1 hr/week, > 1 hr/week) at ages 4 and 7 years. Child sex and exact age were included in all crude and adjusted statistical models. To evaluate whether the assumed relationships and the minimum adjustment sets provided by the DAGs are supported by our data, we conducted forward and backward 10% change-in-estimate procedures departing from the minimum adjustment sets following the methods suggested by Evans et al. (2012). The overall DAG of the assumed or known causal relationships between covariates included in the final models is shown in Supplemental Material, Figure S1.

Few covariates had > 3% of missing values; only information on dietary habits at age 4 years was missing in 11–12%. To maximize the sample size, we created missing categories in potential confounders. Results of complete-case analyses with missing observations excluded (data not shown) were similar to results using missing categories.

In sensitivity analyses we tested associations in multi-pollutant models simultaneously adjusted for ΣHMWPm and ΣLMWPm. We further repeated analyses excluding *a*) preterm births (i.e., < 37 weeks of gestation, *n* ≤ 8, depending on the outcome model) because they may follow different catch-up growth trajectories (Euser et al. 2008); *b*) mother–child pairs with gestational diabetes (*n* ≤ 6); *c*) mother–child pairs with very diluted urine samples in pregnancy (i.e., creatinine < 0.3 g/L, *n* ≤ 30); and *d*) outliers of phthalate metabolite concentrations (0.5–2% of mothers with the highest concentrations depending on metabolite). We also repeated analysis using phthalate metabolite concentrations (nanograms per milliliter) and including urine creatinine levels as a separate covariate in the models.

DAGs were drawn using the DAGitty version 2.0 (Textor et al. 2011). Analyses were conducted by using STATA software, version 10 (StataCorp).

## Results

*Description of population characteristics*. Twenty-five percent of children from birth to 6 months of age were rapid growers and 22–29% were overweight at 1, 4, and 7 years of age (Table 1). The waist-to-height ratio was elevated in 33% and 21% of children at ages 4 and 7 years, respectively. Rapid growth and overweight were more prevalent in boys compared with girls. Sex differences in additional maternal or child covariates were not observed (Table 1). Children included in analysis did not differ from those excluded (due to missing information in phthalate determinations) with respect to the study outcome variables, but their mothers were more likely to have higher education (university: 35% vs. 19%) and higher social class (professionals and managers: 24% vs. 12%), and were less likely to have smoked during pregnancy (27% vs. 36%).

**Table 1:** Main characteristics of mother–child pairs in the analysis population.

Characteristic	All (% or mean ± SD)	Girls*ª* (% or mean ± SD)	Boys*ª* (% or mean ± SD)
Child characteristics
From birth to 6 months of age	*n* = 391	*n* = 186	*n* = 205
Gestational age (weeks)	39.7 ± 1.4
Birth weight (g)	3,264 ± 394	3,202 ± 403	3,322 ± 378
Birth weight *z*-score (SD)	–0.1 ± 0.8
Birth length (cm)	49.4 ± 1.8	49.0 ± 1.8	49.8 ± 1.9
Weight at 6 months (g)	7,654 ± 860	7,337 ± 776	7,937 ± 833
Weight *z*-score at 6 months (SD)	–0.06 ± 0.9
Weight gain *z*-score 0–6 months (SD)	0.05 ± 0.1
Rapid growth 0–6 months, yes (%)	25	23	27
Exclusive breastfeeding ≥ 16 weeks (%)	49
At 1 year of age	*n* = 377	*n* = 178	*n* = 199
Exact age (months)	14.5 ± 0.7
Weight (kg)	10.3 ± 1.2	9.9 ± 1.1	10.6 ± 1.1
Length (cm)	77.5 ± 2.8	76.8 ± 2.8	78.2 ± 2.7
BMI *z*-score (SD)	0.5 ± 0.9	0.5 ± 0.8	0.6 ± 0.9
BMI *z*-score ≥ 85th percentile (%)	27	23	30
At 4 years of age	*n* = 370	*n* = 176	*n* = 194
Exact age (months)	53.3 ± 2.1
Weight (kg)	18.1 ± 2.8	17.8 ± 2.8	18.4 ± 2.8
Height (cm)	106 ± 4.5	105 ± 4.7	106 ± 4.2
BMI *z*-score (SD)	0.51 ± 1.0	0.37 ± 0.9	0.63 ± 1.1
BMI *z*-score ≥ 85th percentile (%)	22	19	25
Waist circumference (cm)^*b*^	52 ± 5.1
Waist-to-height ratio^*b*^	0.49 ± 0.04
Waist-to-height ratio > 0.50 (%)^*b*^	33	35	31
Systolic BP (mmHg)^*c*^	101 ± 12	100 ± 12	102 ± 12
Diastolic BP (mmHg)^*c*^	65 ± 12	64 ± 10	66 ± 13
At 7 years of age	*n* = 360	*n* = 171	*n* = 189
Exact age (months)	82 ± 4.9
Weight (kg)	25.1 ± 5.2	24.6 ± 5.3	25.4 ± 5.1
Height (cm)	121 ± 5.8	121 ± 5.9	122 ± 5.6
BMI *z*-score (SD)	0.68 ± 1.2	0.57 ± 1.1	0.78 ± 1.3
BMI *z*-score ≥ 85th percentile (%)	29	26	32
Waist circumference (cm)	57.6 ± 6.8
Waist-to-height ratio	0.47 ± 0.05
Waist-to-height ratio > 0.50 (%)	21	22	21
Systolic BP (mmHg)^*d*^	105 ± 10	104 ± 11	106 ± 10
Diastolic BP (mmHg)^*d*^	63 ± 10	63 ± 11	62 ± 8
Maternal characteristics	*n* = 391
Country of origin (%)
Spain	93
Other	6
Missing	1
Age at delivery (years)	30.6 ± 4.0
Education (%)
Primary	22
Secondary	43
University	35
Social class (ISCO-88 code) (%)
Professionals, managers (I, II)	24
Other nonmanual (III)	32
Skilled, semiskilled, unskilled manual (IV, V)	44
Parity, nulliparous (%)	56
Prepregnancy BMI (kg/m^2^)	23.7 ± 4.6
Smoking (%)
Never/stopped before pregnancy	73
Yes, until 1st pregnancy trimester	13
Yes, throughout pregnancy	14
^***a***^Characteristics that differ according to sex (*p* ≤ 0.20) are shown separately for girls and boys. Chi-square test was used to test for categorical variables, and *t*-test for continuous variables (all continuous variables normally distributed). ^***b***^*n* = 366 (174 girls, 192 boys) due to missing numbers in the waist circumference covariate. ^***c***^*n* = 342 (169 girls, 173 boys) due to missing numbers in the BP covariates. ^***d***^*n* = 355 (170 girls, 185 boys) due to missing numbers in the BP covariates.			

Pearson correlation coefficients between the different child ages at examination were in the range of 0.46–0.82 for BMI *z*-scores, 0.56 for waist-to-height ratio, and 0.13 for systolic BP *z*-scores, and no correlation was shown for diastolic BP *z*-scores (Pearson coefficient = 0.00). Within-age correlation coefficients of the different outcome variables ranged from 0.07 to 0.67 at 4 years and from 0.21 to 0.89 at 7 years of age; at both ages, correlations were lowest between BMI and diastolic BP *z*-scores and highest between BMI *z*-scores and waist-to-height ratio (data not shown).

The phthalate metabolite with the highest concentrations in maternal urine was MEP (Table 2). The Pearson correlation coefficient between the ΣHMWPm and the ΣLMWPm was 0.18. Phthalate metabolite concentrations (individual and sums) were linearly related to all outcome variables (GAM *p*-gain > 0.10), except from systolic and diastolic BP *z*-scores for which nonlinear relationships (GAM *p*-gain < 0.05) were generally observed in girls and boys for both ΣHMWPm and ΣLMWPm (data not shown). Thus, tertile-specific effect estimates are shown hereafter for BP *z*-scores, and effect estimates per doubling of phthalate metabolite concentrations (i.e., log_2_-transformed) for all other outcomes.

**Table 2:** Average first- through third-trimester concentrations of phthalate metabolites (μg/g creatinine) in the INMA-Sabadell birth cohort (*n* = 391).*^a^*

Parent phthalate	Metabolite	GM (GSD)^*b*^	Minimum	Percentile			Maximum
				25th	50th	75th	
DEHP	MEHP	11.4 (2.0)	1.8	7.3	11.0	17.2	267
DEHP	MEHHP	29.1 (2.0)	5.3	17.9	28.0	41.5	503
DEHP	MEOHP	21.7 (1.9)	4.1	14.3	20.8	30.3	378
DEHP	MECPP	41.6 (1.9)	14.4	31.0	42.5	61.2	1,086
	ΣDEHPm	99.6 (1.8)	24.8	64.9	95.1	139	1,554
BBzP	MBzP	12.6 (2.2)	1.5	7.1	11.9	20.1	405
	ΣHMWPm	118 (1.8)	33.4	79.7	112	159	1,868
DEP	MEP	389 (2.6)	34.0	199	405	804	9,379
DiBP	MiBP	33.0 (1.9)	5.1	21.6	31.4	48.2	334
DnBP	MnBP^*c*^	32.7 (2.1)	5.8	19.9	30.7	47.3	836
	ΣLMWPm	482 (2.3)	62.3	258	472	851	9,948
^***a***^Phthalate metabolite concentrations are shown after substituting the < LOD values by LOD/2. Values < LOD for all phthalate metabolites were ≤ 1% in all the first- and third-trimester urine samples analyzed (Valvi et al. 2015). ^***b***^Creatinine concentrations geometric mean (GM) [geometric standard deviation (GSD)] = 0.8 (1.8) and 0.9 (1.7) in first and third trimester, respectively. ^***c***^Small amounts may also be due to metabolism of BBzP.							

*HMWP metabolites and growth and BP outcomes*. The ΣHMWPm was associated with a lower weight gain *z*-score in the first 6 months of age in boys [adjusted β = –0.41; 95% confidence interval (CI): –0.75, –0.06] and with somewhat higher weight gain *z*-score in girls (adjusted β = 0.24; 95% CI: –0.16, 0.65) (*p*-sex interaction = 0.01) (Table 3). The ΣHMWPm was associated with lower BMI *z*-scores in boys at any age (adjusted β = –0.28; 95% CI: –0.60, 0.03) and with higher BMI *z*-scores in girls (adjusted β = 0.30; 95% CI: –0.04, 0.64) (*p*-sex interaction = 0.05). There was some suggestion that child age may influence the associations with BMI *z*-scores in boys with significant negative associations observed at 4 and 7 years of age and also a negative but nonsignificant association shown at 1 year of age (*p*-age interaction = 0.10 at 4 years and 0.11 at 7 years of age). In girls, associations at each age were positive but not statistically significant (*p*-age interaction = 0.82 at 4 years and 0.58 at 7 years of age) (Table 3). No association was observed with waist-to-height ratio in either boys or girls. The ΣHMWPm was associated with significantly lower systolic BP *z*-scores in girls for all ages combined (adjusted β = –0.39; 95% CI: –0.65, –0.12 for the 2nd tertile and –0.28; –0.55, –0.01 for the 3rd tertile of exposure), but associations were not significant in boys (*p*-sex interaction = 0.11 and 0.10 for the 2nd and 3rd tertiles of exposure, respectively). There was no evidence of modification by child age (*p*-age interaction ≥ 0.37 in the overall and sex-specific models). Adjusting the models for child BMI *z*-scores did not change the associations between ΣHMWPm and systolic BP *z*-scores in either sex (data not shown). There was no consistent evidence of an association between ΣHMWPm and diastolic BP *z*-scores in either sex. When we analysed dichotomous outcomes, results for rapid growth and overweight were consistent with those for weight gain and BMI *z*-scores, but none of the relative risks (RRs) reached the level of statistical significance; further, there was no association with any other of the dichotomous outcomes studied (see Supplemental Material, Figure S2A). When ΣDEHPm and MBzP were analyzed separately, both ΣDEHPm and MBzP were associated with lower weight gain *z*-score in boys but not in girls (*p*-sex interaction = 0.03 and 0.07, respectively). The significant negative associations between ΣHMWPm and BMI *z*-scores in boys and between ΣHMWPm and systolic BP *z*-scores in girls were shown to be driven mainly by ΣDEHPm phthalates (see Supplemental Material, Table S1).

**Table 3:** Average first- through third-trimester concentrations of ΣHMWPm metabolites (log_2_-transformed, μg/g creatinine) and growth and BP outcomes.

Outcome/age at examination	All β^*a*^ (95% CI)	Girls β^*a*^ (95% CI)	Boys β^*a*^ (95% CI)	*p*-Sex interaction
Weight gain *z*-score, 0–6 months	*n *= 391	*n *= 186	*n *= 205
	–0.15 (–0.41, 0.11)	0.24 (–0.16, 0.65)	–0.41 (–0.75, –0.06)	0.01
BMI *z*-scores^*b*^	*n *= 391/1,107	*n *= 186/525	*n *= 205/582
All ages	–0.06 (–0.29, 0.17)	0.30 (–0.04, 0.64)	–0.28 (–0.60, 0.03)	0.05
1 year	0.04 (–0.24, 0.31)	0.26 (–0.13, 0.65)	–0.11 (–0.47, 0.26)
4 years	–0.11 (–0.39, 0.16)	0.24 (–0.15, 0.64)	–0.38 (–0.76, –0.01)
7 years	–0.16 (–0.43, 0.12)	0.16 (–0.24, 0.56)	–0.40 (–0.78, –0.02)
Waist-to-height ratio^*b*^	*n *= 382/726	*n *= 182/345	*n *= 200/381
All ages	–0.01 (–0.01, 0.01)	0.00 (–0.01, 0.02)	–0.01 (–0.02, 0.00)	0.39
4 years	–0.01 (–0.02, 0.00)	0.00 (–0.02, 0.02)	–0.01 (–0.02, 0.00)
7 years	–0.00 (–0.01, 0.01)	0.01 (–0.01, 0.03)	–0.01 (–0.02, 0.01)
Systolic BP *z*-scores^*b*^^,^^*c*^	*n *= 379/697	*n *= 181/339	*n *= 198/358
All ages
T2	–0.23 (–0.41, –0.06)	–0.39 (–0.65, –0.12)	–0.14 (–0.37, 0.09)	0.11
T3	–0.14 (–0.34, 0.03)	–0.28 (–0.55, –0.01)	–0.03 (–0.26, 0.20)	0.10
4 years
T2	–0.16 (–0.39, 0.07)	–0.30 (–0.60, –0.01)	–0.01 (–0.34, 0.32)
T3	–0.17 (–0.41, 0.06)	–0.32 (–0.62, –0.03)	–0.06 (–0.39, 0.26)
7 years
T2	–0.30 (–0.53, –0.08)	–0.45 (–0.74, –0.16)	–0.25 (–0.56, 0.06)
T3	–0.14 (–0.37, 0.09)	–0.29 (–0.58, 0.00)	0.01 (–0.30, 0.33)
Diastolic BP *z*-scores^*b*^^,^^*c*^	*n *= 379/697	*n *= 181/339	*n *= 198/358
All ages
T2	0.07 (–0.10, 0.23)	–0.02 (–0.26, 0.23)	0.13 (–0.09, 0.36)	0.25
T3	0.01 (–0.15, 0.18)	0.02 (–0.23, 0.27)	0.01 (–0.21, 0.23)	0.89
4 years
T2	0.16 (–0.08, 0.39)	–0.03 (–0.37, 0.30)	0.34 (0.01, 0.66)
T3	0.05 (–0.18, 0.28)	0.01 (–0.33, 0.35)	0.10 (–0.22, 0.42)
7 years
T2	–0.03 (–0.26, 0.20)	–0.02 (–0.36, 0.31)	–0.04 (–0.35, 0.27)
T3	–0.05 (–0.28, 0.17)	–0.04 (–0.38, 0.29)	–0.05 (–0.36, 0.26)
^***a***^All models were adjusted for child sex, exact age at examination, and maternal characteristics (country of origin, age at delivery, parity, education, social class, prepregnancy BMI, and smoking in pregnancy). ^***b***^Coefficients were estimated by GEE models; *n* represents numbers of individuals/outcome measures. ^***c***^Effect estimates are shown per tertile of exposure (T2, tertile 2; T3, tertile 3) due to nonlinear associations between the exposure and the outcome. The β in the reference groups is 0.				

*LMWP metabolites and growth and BP outcomes*. The ΣLMWPm was not significantly associated with weight gain, BMI *z*-scores, or waist-to-height ratio at any age (Table 4). The ΣLMWPm was associated with lower systolic BP *z*-scores in girls (adjusted β = –0.23; 95% CI: –0.50, –0.04 in 2nd tertile and –0.40; –0.66, –0.12 in 3rd tertile of exposure) but not in boys (*p*-sex interaction = 0.17 for the 2nd and < 0.01 in the 3rd tertile of exposure). Child age was not shown to influence these associations overall or in girls and boys separately (*p*-age interaction ≥ 0.25 at 4 and 7 years of age). Associations between the ΣLMWPm and diastolic BP *z*-scores were negative at all ages, overall and in girls or boys separately, but none of the associations reached the level of statistical significance. When we analyzed dichotomous outcomes, there was no association with any of the growth or BP outcomes studied overall or in boys and girls separately (see Supplemental Material, Figure S2B). From the individual LMWP metabolites analyzed, only MEP was significantly associated with lower systolic BP *z*-scores in girls; associations for MiBP and MnBP were in the same direction but did not reach the level of statistical significance (see Supplemental Material, Table S2). Individual LMWP were not significantly associated with any other outcome studied.

**Table 4:** Average first- through third-trimester concentrations of ΣLMWPm metabolites (log_2_-transformed, μg/g creatinine) and growth and BP outcomes.

Outcome/age at examination	All β^*a*^ (95% CI)	Girls β^*a*^ (95% CI)	Boys β^*a*^ (95% CI)	*p*-Sex interaction
Weight gain *z*-score 0–6 months	*n *= 391	*n *= 186	*n *= 205
	0.03 (–0.16, 0.23)	–0.06 (–0.34, 0.22)	0.14 (–0.14, 0.42)	0.38
BMI *z*-scores^*b*^	*n *= 391/1,107	*n *= 186/525	*n *= 205/582
All ages	0.08 (–0.13, 0.86)	0.03 (–0.20, 0.26)	0.12 (–0.12, 0.37)	0.30
1 year	0.05 (–0.14, 0.25)	0.11 (–0.16, 0.38)	0.01 (–0.27, 0.29)
4 years	0.09 (–0.11, 0.29)	0.04 (–0.24, 0.31)	0.17 (–0.12, 0.45)
7 years	0.12 (–0.08, 0.32)	0.00 (–0.28, 0.28)	0.21 (–0.08, 0.50)
Waist-to-height ratio^*b*^	*n *= 382/726	*n *= 182/345	*n *= 200/381
All ages	0.00 (–0.01, 0.01)	0.00 (–0.01, 0.01)	0.01 (–0.01, 0.02)	0.41
4 years	0.00 (–0.01, 0.01)	0.00 (–0.01, 0.02)	0.00 (–0.01, 0.01)
7 years	0.01 (–0.01, 0.01)	0.00 (–0.01, 0.02)	0.01 (–0.01, 0.02)
Systolic BP *z*-scores^*b*^^,^^*c*^	*n *= 379/697	*n *= 181/339	*n *= 198/358
All ages
T2	–0.05 (–0.23, 0.12)	–0.23 (–0.50, 0.04)	0.05 (–0.17, 0.28)	0.17
T3	–0.13 (–0.31, 0.05)	–0.40 (–0.66, –0.12)	0.12 (–0.11, 0.35)	< 0.01
4 years
T2	–0.09 (–0.32, 0.14)	–0.24 (–0.57, 0.09)	0.02 (–0.30, 0.34)
T3	–0.20 (–0.44, 0.03)	–0.38 (–0.72, –0.05)	0.01 (–0.32, 0.34)
7 years
T2	–0.02 (–0.25, 0.21)	–0.22 (–0.56, 0.12)	0.08 (–0.23, 0.39)
T3	–0.06 (–0.30, 0.16)	–0.43 (–0.77, –0.09)	0.25 (–0.07, 0.57)
Diastolic BP *z*-scores^*b*^^,^^*c*^	*n *= 379/697	*n *= 181/339	*n *= 198/358
All ages
T2	–0.12 (–0.28, 0.04)	–0.19 (–0.44, 0.06)	–0.08 (–0.30, 0.14)	0.48
T3	–0.14 (–0.31, 0.02)	–0.17 (–0.44, 0.06)	–0.12 (–0.34, 0.11)	0.72
4 years
T2	–0.17 (–0.40, 0.06)	–0.27 (–0.60, 0.07)	–0.10 (–0.42, 0.23)
T3	–0.14 (–0.37, 0.10)	–0.06 (–0.40, 0.27)	–0.20 (–0.53, 0.13)
7 years
T2	–0.08 (–0.31, 0.15)	–0.12 (–0.46, 0.21)	–0.09 (–0.39, 0.22)
T3	–0.13 (–0.37, 0.10)	–0.29 (–0.63, 0.05)	–0.01 (–0.29, 0.34)
^***a***^All models were adjusted for child sex, exact age at examination, and maternal characteristics (country of origin, age at delivery, parity, education, social class, prepregnancy BMI, and smoking in pregnancy). ^***b***^Coefficients were estimated by GEE models; *n* represents numbers of individuals/outcome measures. ^***c***^Effect estimates are shown per tertile of exposure (T2, tertile 2; T3, tertile 3) due to nonlinear associations between the exposure and the outcome. The β in the reference groups is 0.				

*Sensitivity analyses*. The magnitude and statistical significance of the associations between ΣHMWPm and ΣLMWPm and the growth and BP outcomes did not change in the multi-pollutant models adjusted for both ΣHMWPm and ΣLMWPm (data not shown). The exclusions of preterm births, mother–child pairs with gestational diabetes or with diluted urine samples in pregnancy, and few outliers of phthalate metabolite concentrations did not change the associations of interest (data not shown). Repeating analysis including phthalate metabolite concentrations in nanograms per milliliter and creatinine as a separate covariate in the models did not change the direction or statistical significance of the associations (data not shown).

## Discussion

In this prospective study, prenatal exposure to ΣHMWPm was associated with lower weight *z*-score difference in the first 6 months of life and lower BMI *z*-scores in boys at 4–7 years of age. In girls, we found some evidence that ΣHMWPm may be associated with higher BMI *z*-scores; however, associations did not reach the level of statistical significance at any age. ΣHMWPm was not associated with waist-to-height ratio. ΣLMWPm was not significantly associated with any of the growth outcomes in either sex. Both ΣHMWPm and ΣLMWPm were associated with lower systolic BP *z*-scores at 4–7 years of age in girls but not in boys, and no significant association was shown with diastolic BP *z*-scores.

One prospective study in a Danish population (*n* = 61) has recently reported higher cord-blood levels of MEOHP to be associated with reduced BMIs between birth and 11 months of age in boys, whereas in girls the reverse was found (de Cock et al. 2014). Results from our study for DEHP metabolites and growth outcomes are in agreement with this small Danish study, but our findings are inconsistent with those from some previous cross-sectional studies. Associations between sums of HMWP or MEHP separately and BMI in children and/or adolescents were positive in a Chinese population (Wang et al. 2013), negative in adolescent U.S. girls, and negative but not significant in boys (Hatch et al. 2008) and null in both sexes in three other U.S. populations (Buser et al. 2014; Trasande et al. 2013a; Wolff et al. 2010). Further, a cross-sectional study in Danish school-age children reported DEHP urine metabolites to be negatively associated with recent weight and height gain and serum levels of free and total triodothyronine (T_3_) and insulin-like growth factor 1, and the associations with hormone levels were shown to be modified by sex (Boas et al. 2010). The cross-sectional evidence for the associations between MEP or sums of LMWP and BMI in children and/or adolescents is similarly inconsistent with positive (Buser et al. 2014; Trasande et al. 2013b; Wang et al. 2013), not significantly positive in girls and negative in boys (Hatch et al. 2008), negative (Boas et al. 2010), or null (Wolff et al. 2010) associations reported previously. One of these studies reported positive associations in non-Hispanic black children but null associations in Hispanic and non-Hispanic white children, and no effect modification by child sex (Trasande et al. 2013a), whereas one other reported significant associations to be seen only in boys but not in girls (Buser et al. 2014). Our findings cannot be directly compared to those of cross-sectional studies because the effects of prenatal compared with postnatal phthalate exposure on child growth could differ, and exposure assessment in all previous studies was based on one single spot-urine sample increasing the likelihood of exposure misclassification. Further, the existing evidence seems to suggest that effects, if any, may vary according to child age and race. Reverse causation is also more likely in cross-sectional studies because variations in behaviors linked to phthalate exposure such as the consumption of packaged foods and beverages between obese and lean subjects could explain the associations shown.

Although further research is needed to clarify the underlying mechanisms, experimental evidence suggests that developmental exposure to phthalates may impair growth in a sex-specific manner. Developmental effects of phthalate exposure on adipogenesis may be mediated though the disruption of the steroid and/or thyroid hormone molecular pathways and the inappropriate activation of the PPARγ (Boberg et al. 2008; Feige et al. 2010; Gray et al. 2000; Hao et al. 2012; Ishihara et al. 2003; Lin et al. 2011; Shen et al. 2009). Exposures to benzylbutyl (BBzP), DEHP, and their metabolites have been shown to exhibit anti-androgenic effects in male rodents (Boberg et al. 2008; Gray et al. 2000). Phthalates are further suggested to be thyroid receptor antagonists (Shen et al. 2009) and to interfere with the binding of T_3_ to transport proteins (Ishihara et al. 2003). Perinatal MEHP exposure at relatively low levels induced adipogenesis and lipid storage in male mice offspring via inducing the expression and activation of PPARγ receptors in the adipose tissue, but no effects were shown in females (Hao et al. 2012). Some protective effects on weight gain linked to DEHP exposure have also been observed in mice in other studies (Feige et al. 2010; Lin et al. 2011). We found that prenatal HMWP exposure is associated with lower early weight gain in infancy and lower BMI *z*-scores later in childhood, up to 7 years of age in boys, and some suggestion that HMWP exposure may be associated with higher BMI *z*-scores in girls. Continuous follow-up will elucidate whether these associations persist at later ages.

Our findings suggest that prenatal exposure mainly to DEHP metabolites and MEP is associated with lower systolic BP *z*-scores in girls independent of child BMI; however, the recent analysis of the 2003–2008 NHANES survey suggested a 0.04-SD unit increase in systolic BP *z*-score for each 3-fold (roughly) increase in DEHP metabolites in children 6–19 years of age (Trasande et al. 2013b). In this other study, associations appeared to be stronger in boys, younger children, and children who were not overweight, whereas no association was shown with diastolic BP or between LMWP and either systolic or diastolic BP. Inverse associations between MEP and systolic and diastolic BP have been further shown in elderly adults from the Prospective Investigation of the Vasculature in Uppsala Seniors study (Olsén et al. 2012). Exposures to HMWP and LMWP have been associated with increased levels of oxidative stress markers (e.g., 8-hydroxydeoxyguanosine and 8-isoprostane) in urine of pregnant women (Ferguson et al. 2014), and increased oxidative stress during pregnancy is thought to alter the fetal programming of cardiovascular function and to increase the CVD risk in later life (Giussani et al. 2012). Thus, continuous follow-up is required to explore whether the negative associations shown in our study between prenatal phthalate exposure and systolic BP in girls at 4–7 years of age persist or reverse at later ages.

Individual HMWP or LMWP metabolites are moderately to highly correlated to each other (Valvi et al. 2015), so disentangling the effects of individual metabolites in human populations is difficult. We found some evidence that ΣHMWPm associations may be driven mainly by ΣDEHPm and that ΣLMWPm associations may be driven mainly by MEP. However, the effect estimates of the other metabolites included in the sums were in the same direction. Associations of the different individual DEHP metabolites were very similar (data not shown). Although the magnitude of health effects can differ for the different parent and metabolite phthalates, it could also be that the higher concentrations and the wider range of concentrations of DEHP metabolites and MEP compared with the other metabolites has increased the ability for detecting statistically significant associations only for these compounds.

The *z*-score increases for the growth and BP outcomes we estimated were of similar magnitude to those related to maternal smoking during pregnancy, a well-established determinant of childhood cardiometabolic risk (Behl et al. 2013) (β and 95% CI for children whose mothers had smoked throughout pregnancy compared with children of nonsmokers in our final adjusted models was 0.27; –0.01, 0.55 for weight gain *z*-score; 0.30; 0.01, 0.50 for BMI *z*-scores, and 0.31; –0.01, 0.64 for systolic BP *z*-scores). This suggests that the associations shown in this study could be of important clinical relevance and therefore worthy to be further explored in other settings. The urinary phthalate metabolites in this population are on average comparable to those measured in pregnant women in other Spanish regions (Casas et al. 2011) and lower compared with those measured in other European pregnancy cohorts with samples collected in 2000–2006, for all metabolites except from MEP, for which lower levels are reported in a French study (Casas et al. 2013b). The prospective design is a great advantage of our study over the existing literature because it rules out reverse causation and, further, it has permitted evaluation of the associations of prenatal phthalate exposure and repeated growth and BP outcomes at different ages. Although we assessed phthalate exposure using the average of concentrations measured in two spot-urine samples, misclassification is still likely due to the high within-subject variability of phthalate metabolites shown in this and other populations (Valvi et al. 2015). However, a nondifferential error in terms of the outcomes is more likely to have biased effect estimates toward the null (Armstrong 1998). Future studies should aim to optimize exposure assessment by using more than two spot-urine samples. Further, we do not have measurements of phthalate exposure in the children, which would have permitted us to evaluate potential confounding by postnatal exposures and associations at different windows of susceptibility. Limitations in our study related to the outcome assessment are the lack of more direct measurements of fat mass (e.g., dual-energy X-ray absorptiometry) than BMI and waist circumference and the lack of repeated measurements of BP at each child age. We evaluated a wide list of potential confounders and few covariates provided in the initial DAGs were shown to change the effect estimates by > 10%. Further, we found no evidence of substantial inflation in effect estimates when we compared the crude (data not shown) with the final multivariate-adjusted estimates. However, we cannot rule out the possibility that associations may be partially explained by residual confounding. Further, children not included in analysis were shown to be of mothers with lower education and social class, who were more likely to have smoked during pregnancy. Given that these characteristics are shown to increase phthalate exposure in this population (Valvi et al. 2015) and that associations with weight homeostasis have been suggested to be nonmonotonic with different effects shown at high and lower levels of exposure (Hao et al. 2012), our findings may not apply to more disadvantaged groups of the population.

Our study provides evidence that prenatal exposure to phthalates may influence postnatal growth and blood pressure differently in boys and girls up to 7 years of age. The inconsistencies between our findings and those previously shown in some cross-sectional studies highlight the necessity for evaluating these associations in prospective studies.

## Supplemental Material

(915 KB) PDF
