# Supplementary material for: Prenatal Phthalate Exposure and Childhood Growth and Blood Pressure: Evidence from the Spanish INMA-Sabadell Birth Cohort Study
Source: Environ Health Perspect. 2015 Apr 7;123(10):1022–9. doi: 10.1289/ehp.1408887 (PMC4590754; doi:10.1289/ehp.1408887)
Supplement: (915 KB) PDF [file ehp.1408887.s001.acco.pdf]

*Environ Health Perspect*
doi: 10.1289/ehp.1408887

**Note to Readers:** *EHP* strives to ensure that all journal content is accessible to all readers. However, some figures and Supplemental Material published in *EHP* articles may not conform to 508 standards due to the complexity of the information being presented. If you need assistance accessing journal content, please contact ehp508@niehs.nih.gov. Our staff will work with you to assess and meet your accessibility needs within 3 working days.

# Supplemental Material

# Prenatal Phthalate Exposure and Childhood Growth and Blood Pressure: Evidence from the Spanish INMA-Sabadell Birth Cohort Study

Damaskini Valvi, Maribel Casas, Dora Romaguera, Nuria Monfort, Rosa Ventura, David Martinez, Jordi Sunyer, and Martine Vrijheid

## Table of Contents

**Figure S1.** Directed Acyclic Graph of the known or assumed relationships between covariates retained in the final models.

**Figure S2.** Associations between the average 1st-3rd pregnancy trimester concentrations of ΣHMWPm (A) and ΣLMWPm (B) metabolites ($log_2$-transformed, in µg-g creatinine) and dichotomous outcomes. All models adjusted for child sex, exact age at examination and maternal characteristics (country of origin, age at delivery, parity, education, social class, prepregnancy BMI and smoking in pregnancy). Effect estimates for systolic and diastolic BP are shown per tertile of exposure (i.e. T2: tertile 2; T3: tertile 3). The RR in the reference groups is 1. (Phthalate exposure modeled continuously for all other outcomes).

**Table S1.** Average 1st-3rd pregnancy trimester concentrations of ΣDEHPm and MBzP metabolites ($log_2$-transformed, in µg-g creatinine) and growth and BP outcomes.

**Table S2.** Average 1st-3rd pregnancy trimester concentrations of MEP, MiBP and MnBP metabolites ($log_2$-transformed, in µg-g creatinine) and BP outcomes.

**Figure S1.** Directed Acyclic Graph of the known or assumed relationships between covariates retained in the final models.

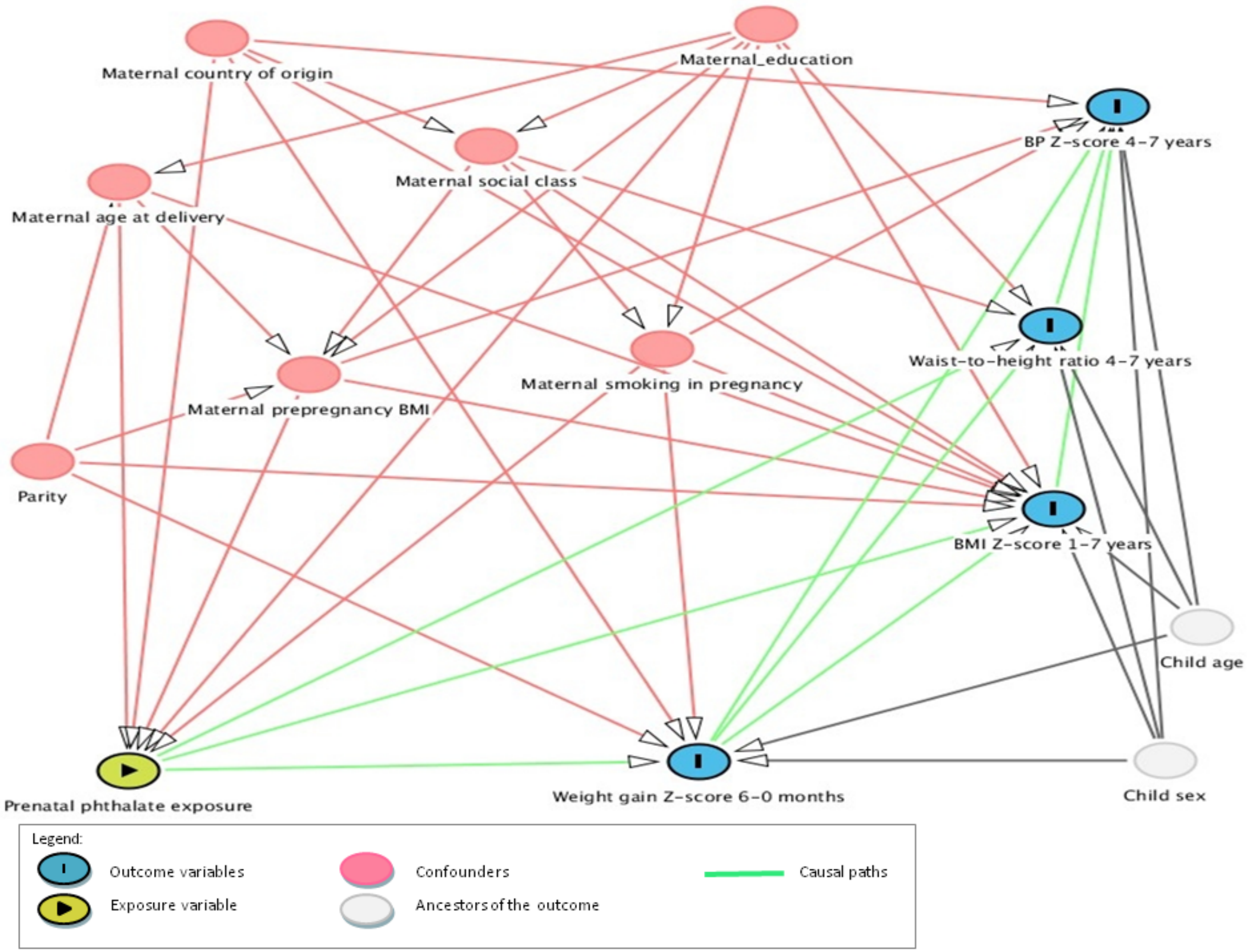

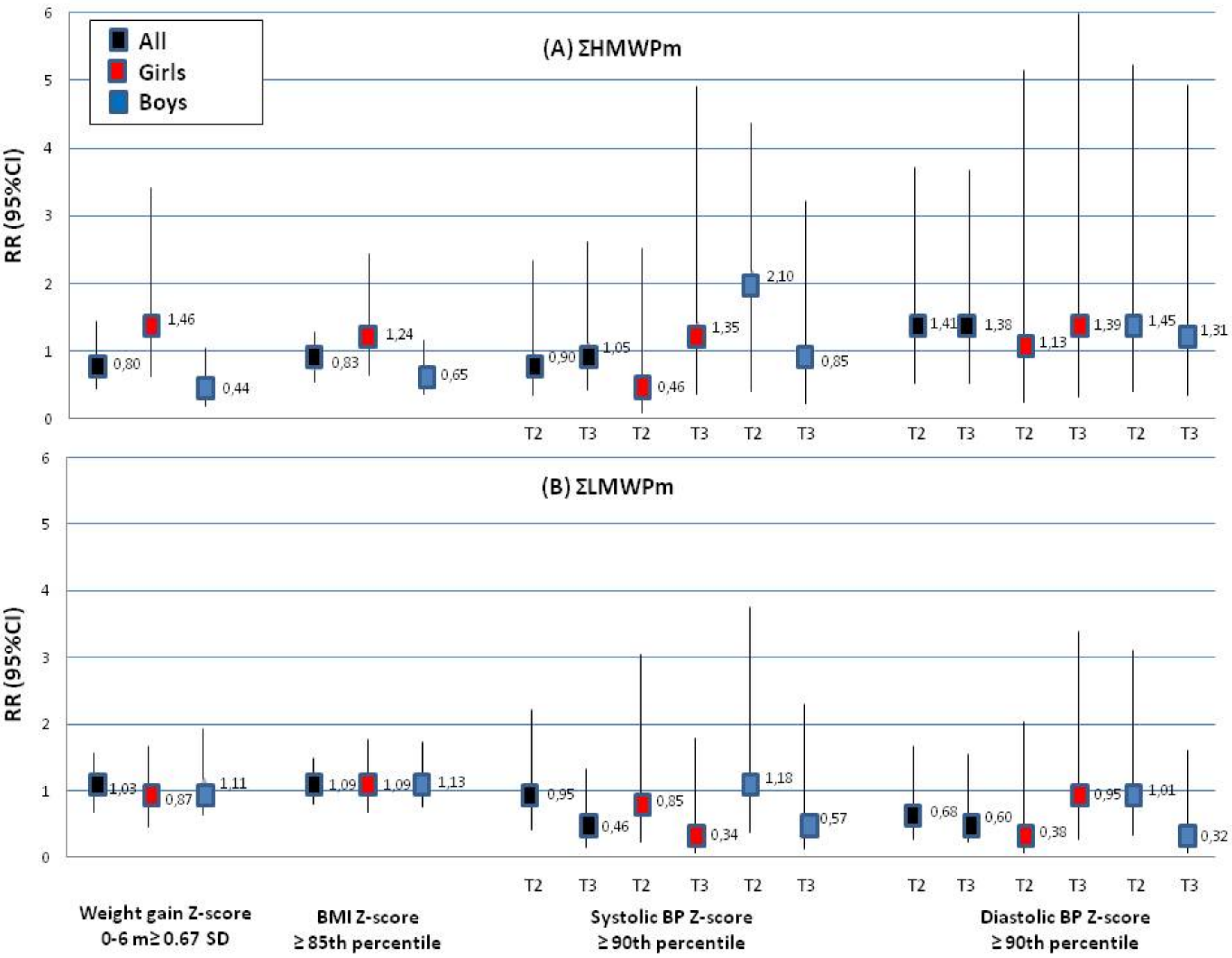


**Figure S2.** Associations between the average 1st-3rd pregnancy trimester concentrations of ΣHMWPm (A) and ΣLMWPm (B) metabolites ($log_2$-transformed, in µg-g creatinine) and dichotomous outcomes. All models adjusted for child sex, exact age at examination and maternal characteristics (country of origin, age at delivery, parity, education, social class, prepregnancy BMI and smoking in pregnancy). Effect estimates for systolic and diastolic BP are shown per tertile of exposure (i.e. T2: tertile 2; T3: tertile 3). The RR in the reference groups is 1. (Phthalate exposure modeled continuously for all other outcomes).

**Table S1.** Average 1st-3rd pregnancy trimester concentrations of ΣDEHPm and MBzP metabolites ($log_2$-transformed, in μg-g creatinine) and growth and BP outcomes.

| **Outcome/Age at examination** | | **ΣDEHPm**[a] | | | **MBzP**[a] | | |
|---|---|---|---|---|---|---|---|
| | | **Girls β (95% CI)** | **Boys β (95% CI)** | **P-sex interaction** | **Girls β (95% CI)** | **Boys β (95% CI)** | **P-sex interaction** |
| Weight gain Z-score 0-6 months | | n=186 | n=205 | | n=186 | n=205 | |
| | | 0.26 (-0.13, 0.65) | -0.36 (-0.70, -0.01) | 0.01 | 0.02 (-0.28, 0.31) | -0.28 (-0.55, -0.01) | 0.09 |
| BMI Z-score[b] | | n=186/525 | n=205/582 | | n=186/525 | n=205/582 | |
| All ages | | 0.21 (-0.11, 0.53) | -0.32 (-0.64, -0.02) | 0.03 | 0.10 (-0.16, 0.38) | -0.16 (-0.33, 0.15) | 0.76 |
| Systolic BP Z-score[b,c] | | n=181/339 | n=198/358 | | n=181/339 | n=198/358 | |
| All ages | T2 | -0.50 (-0.77, -0.24) | -0.01 (-0.24, 0.21) | 0.01 | -0.05 (-0.29, 0.25) | -0.10 (-0.34, 0.13) | 0.41 |
| | T3 | -0.37 (-0.63, -0.10) | -0.05 (-0.29, 0.18) | 0.10 | -0.16 (-0.40, 0.10) | -0.15 (-0.37, 0.07) | 0.45 |
| Diastolic BP Z-score[b,c] | | n=181/339 | n=198/358 | | n=181/339 | n=198/358 | |
| All ages | T2 | -0.10 (-0.34, 0.15) | 0.13 (-0.10, 0.34) | 0.44 | 0.03 (-0.19, 0.29) | -0.02 (-0.36, 0.26) | 0.62 |
| | T3 | -0.08 (-0.33, 0.17) | 0.05 (-0.17, 0.27) | 0.93 | -0.05 (-0.17, 0.27) | 0.16 (-0.15, 0.47) | 0.45 |

All models adjusted for child sex, exact age at examination and maternal characteristics (country of origin, age at delivery, parity, education, social class, prepregnancy BMI and smoking in pregnancy).

[a]Pearson correlation coefficients ranged from 0.68 to 0.97 for pairs of metabolites included in ΣDEHPm and from 0.17 to 0.32 for pairs of DEHP metabolites and MBzP. [b]Coefficients estimated by GEE models. N represents numbers of individuals/outcome measures. [c]Effect estimates shown per tertile of exposure (i.e. T2: tertile 2; T3: tertile 3). The *β* in the reference groups is 0.

**Table S2.** Average 1[st]-3[rd] pregnancy trimester concentrations of MEP, MiBP and MnBP metabolites ($log_2$-transformed, in µg-g creatinine) and BP outcomes.

| **Outcome/Age at examination** | | **MEP**[a] | | **MiBP**[a] | | **MnBP**[a] | |
|---|---|---|---|---|---|---|---|
| | | **Girls** **β (95% CI)** | **Boys** **β (95% CI)** | **Girls** **β (95% CI)** | **Boys** **β (95% CI)** | **Girls** **β (95% CI)** | **Boys** **β (95% CI)** |
| Weight gain Z-score 0-6 months | | n=186 | n=205 | n=186 | n=205 | n=186 | n=205 |
| | | -0.05 (-0.29, 0.18) | 0.09 (-0.15, 0.34) | 0.09 (-0.27, 0.46) | -0.18 (-0.53, 0.15) | -0.14 (-0.42, 0.13) | -0.13 (-0.46, 0.21) |
| BMI Z-score[b] | | n=186/525 | n=205/582 | n=186/525 | n=205/582 | n=186/525 | n=205/582 |
| All ages | | 0.08 (-0.16, 0.31) | 0.01 (-0.24, 0.26) | -0.01 (-0.38, 0.35) | -0.11 (-0.47, 0.26) | -0.11 (-0.39, 0.18) | 0.03 (-0.31, 0.38) |
| | | | | | | | |
| Systolic BP Z-score[b,c] | | n=181/339 | n=198/358 | n=181/339 | n=198/358 | n=181/339 | n=198/358 |
| All ages | T2 | -0.15 (-0.41, 0.12) | 0.04 (-0.19, 0.28) | -0.10 (-0.45, 0.15) | 0.08 (-0.17, 0.31) | -0.12 (-0.40, 0.16) | 0.02 (-0.22, 0.26) |
| | T3 | -0.44 (-0.71, -0.18)** | 0.20 (-0.06, 0.44)** | -0.11 (-0.51, 0.11) | 0.07 (-0.27, 0.21) | -0.08 (-0.42, 0.23) | -0.01 (-0.27, 0.21) |
| Diastolic BP Z-score[b,c] | | n=181/339 | n=198/358 | n=181/339 | n=198/358 | n=181/339 | n=198/358 |
| All ages | T2 | -0.17 (-0.42, 0.08) | -0.01 (-0.23, 0.22) | -0.06 (-0.30, 0.29) | 0.20 (-0.07, 0.40) | -0.10(-0.36, 0.10) | -0.06 (-0.19 0.20) |
| | T3 | -0.23 (-0.48, 0.04) | -0.07 (-0.30, 0.16) | 0.03 (-0.19, 0.30) | 0.12 (-0.12, 0.35) | -0.05 (-0.22, 0.20) | -0.00 (-0.26, 0.20) |

All models adjusted for child sex, exact age at examination and maternal characteristics (country of origin, age at delivery, parity, education, social class, prepregnancy BMI and smoking in pregnancy).

[a]Pearson correlation coefficients: 0.12 for MEP-MiBP, 0.15 for MEP-MnBP and 0.46 for MiBP-MnBP. [b]Coefficients estimated by GEE models. N represents numbers of individuals/outcome measures. [c]Effect estimates shown per tertile of exposure (i.e. T2: tertile 2; T3: tertile 3). The $\beta$ in the reference groups is 0. P-value for the interaction term between exposure and child age at examination >0.25 for all models shown in this table.

P-sex interaction: **<0.05.
